# Therapeutic Effect of Sunflower Seeds and Flax Seeds on Diabetes

**DOI:** 10.7759/cureus.17256

**Published:** 2021-08-17

**Authors:** Abdul Rehman, Aamir Saeed, Rabia Kanwal, Sheraz Ahmad, Shabbar H Changazi

**Affiliations:** 1 Medicine, Services Hospital Lahore, Lahore, PAK; 2 Diet and Nutrition, University of Lahore, Lahore, PAK; 3 Orthopaedics, Services Hospital Lahore, Lahore, PAK; 4 Surgery, Services Hospital Lahore, Lahore, PAK

**Keywords:** diabetes, sunflower seeds, flax seeds, chlorogenic acid, secoisolariciresinol diglucosoid

## Abstract

Diabetes mellitus (DM) is a metabolic syndrome that is spreading like an epidemic throughout the world without any differentiation of races and ethnic groups and has become the cause of death worldwide. It is characterized by high levels of glucose in the blood and has different types classified on the basis of varying pathophysiology. Type 1 diabetes or insulin-dependent diabetes is characterized by insulin insufficiency due to autoimmune dysfunction. Type 2 diabetes or non-insulin-dependent diabetes results from the combination of resistance to insulin action or/and inadequate insulin secretion. Gestational diabetes (GDM) is defined as hyperglycemia due to insulin resistance during pregnancy. Other types include the monogenic type of DM such as neonatal diabetes mellitus (NDM), maturity-onset diabetes of young (MODY), and diabetes in metabolic syndrome. Diabetes is diagnosed by criteria given by American Diabetes Association (ADA) for different tests like fasting plasma glucose test and hemoglobin A1c test. It is characterized by polydipsia, polyphagia, hyperglycemia, and glucosuria. Diabetes mellitus is managed through medications but many studies have proven that consumption of particular foods leads to decreased glucose levels in diabetic patients. Seeds like sunflower and flax seeds have a role in the reduction of glucose levels and can be used to treat type 2 diabetes. The bioactive components in these seeds like chlorogenic acid in sunflower seeds and secoisolariciresinol diglucosoid are involved in the treatment of insulin resistance or insulin production. In different studies, different amounts of these seed extracts were consumed by rats and humans and it resulted in better glycemic control, which provides information that these seeds have anti-diabetic properties.

## Introduction and background

Diabetes mellitus (DM) is generally a complex disease that affects all races and ethnic groups due to improper functioning of insulin, secretion of insulin, or can be a result of both and is the major cause of mortality of death worldwide. When insulin is unable to perform its function, blood glucose level rises above the normal levels and the condition called hyperglycemia takes place, which is known as diabetes mellitus. It is recognized by hyperglycemia, polyphagia, polydipsia, and polyuria. These symptoms lead to the diagnosis of diabetes by different tests [1].

People in developed as well as in developing countries have faced the largest epidemics and diabetes mellitus is one of them. The major influence of this disease is seen in South East Asian countries, and globally the number of diabetic patients is increasing rapidly. According to the global estimate by International Diabetes Federation (IDF), 415 million people were suffering from diabetes and the number will increase to 642 million by 2040 [2]. A similar estimate of 422 million people with this syndrome in 2014 was given by WHO and NCD Risk Factor Collaboration. In 2017, the prevalence of diabetes mellitus was 425 million. Its prevalence increases with an increase in age. Almost 25% of people above the age of 65 years suffer from DM. Diabetes can be managed through diet and it was first time discussed by John Rollo in 1797 [3].

Glycated hemoglobin (HbA1c) is a tool used for assessing the long-term glycemic state in diabetic patients. This test was introduced and has been used in clinical practice since the 1980s. It is a chemical linkage between hemoglobin and glucose and the formation of this linkage is indicative of excessive sugar present in the bloodstream that indicates diabetes. It reflects plasma glucose of the past 2-3 months and can be done at any time of the day. The person does not have to fast on that particular day, that is why this test is preferable over other tests. The cut-off point for HbA1c is ≥ 6.5% recommended by World Health Organization. Impaired fasting glucose is another test performed for diagnosing diabetes and the cut-off point for this test is 110mg/dL [4].

Diabetes is classified into different types including insulin-dependent diabetes mellitus (Type 1), non-insulin diabetes mellitus (Type 2), gestational diabetes mellitus (GDM), neonatal diabetes mellitus (NDM), and the maturity-onset diabetes of the young (MODY).

Treating different diseases by consuming food and its extract have fewer side effects than taking chemically prepared medicines. These foods can reduce and treat the severity of a disease such as diabetes. Different seeds like sunflower seeds, flax seeds, or pumpkin seeds are used for treating type 2 diabetes. Seeds contain different micro- and macronutrients along with other bioactive compounds that play role in the regulation of glucose and insulin metabolism [5-9].

In this article, we review studies to determine the effect of sunflower seeds and flax seeds and on type 2 diabetes. Intake of these seeds' extract may reduce insulin resistance in pre-diabetic persons and decreases the risk of developing type 2 diabetes. They contain polyunsaturated fatty acids (PUFA) so they can be recommended as a natural remedy to treat type 2 diabetes as they increase the insulin levels that ultimately reduce the blood glucose levels.

## Review

Effect of sunflower seed (*Helianthus annuus*) extracts on diabetes

Sunflower seeds are cultivated and consumed globally and contain highly nutritious components like fiber, protein, unsaturated fats, selenium, copper, zinc, iron, vitamin E and have several other nutrients, antioxidants, minerals, and vitamins that play a vital role in the prevention or treatment of certain diseases, and diabetes is one of them. These seeds possess anti-diabetic properties due to the presence of chlorogenic acid, quinic acid, caffeic acid, glycosides, and phytosterols. They also contain 20% of proteins that provide sulfur and nitrogen so these sulfur-rich proteins are ideal for human consumption as they help to carry out their metabiological functions such as muscular cell development and insulin production.

Isomers of conjugated linoleic acid like cis-9 and trans-10 present in these seeds are involved in the normalization of impaired glucose tolerance in human being as well as in animals. Natural anti-glycatives and anti-oxidants are more beneficial for treating and preventing diabetes by inhibiting the functions of oxygen-reactive species that play a role in inducing pathways that ultimately lead to diabetes. Advanced glycation end products (AGEs) regenerate when non-enzymatic glycation occurs between sugar and protein, and when accumulated, they are the major contributors to diabetes under hyperglycemic conditions; the sprouts of sunflower have a defensive role against these AGEs. The AGE-inhibitory rate of sunflower seeds at 1mg/mL concentration is 83.29% [10, 11].

A study was performed to know the effect of sunflower sprout on diabetes. The results indicated that the active ingredient in *H. annuus *possesses anti-glycative properties and is present in high levels, so it is good for people with hyperglycemia and with high lipid levels. Studies have proved the anti-diabetic effect of sunflower seed extracts. Another study was performed on rats to determine the anti-diabetic effect of sunflower seeds extracts. In this study, the rats were treated with *H. annuus* extract (HAE) (250mg/kg and 500mg/kg) for 21 days. As a result, there was improved liver glycogen, representing that the rats with diabetic state got better by the consumption of this extract. It was assumed that the anti-diabetic effect of HAE is due to an increase in insulin secretion from pancreatic beta cells or the release of insulin from its bound form. Chlorogenic acid and caffeic acid are present as 70% of total sunflower polyphenols. So chlorogenic acid present in the extract is the inhibitor of glucose-6-phosphatase translocase which converts glucose-6-phosphate to glucose, which results in less generation of glucose, thus reducing the severity of diabetes. So it has an anti-diabetic and anti-oxidant effect [12, 13].

Effect of flaxseeds on diabetes

Flaxseeds consumption on daily basis helps in better glycemic control in pre-diabetic overweight males as well as females. Flaxseeds play role in treating diabetes type 2 as it decreases the fasting plasma glucose (FPG) concentrations in pre-diabetes. According to a study by Hutchins, when 13g/day flaxseeds in low-dose treatment are given, it reduces the fasting plasma glucose. Insulin resistance and the complications that arise due to it can be alleviated by the consumption of low glycemic index foods [14].In another study, a low dose of 20g/day for three months was given to an individual. It significantly reduced the levels of FPG concentrations and insulin resistance, and insulin sensitivity increased by the low-dose treatment of flaxseeds [15].

Flaxseed and its components have an anti-diabetic effect. Glycemic control is improved by flaxseeds and flax lignin. Secoisolariciresinol diglucoside (SDG), the major lignan of flaxseed, and flax lignan complex are components of flaxseeds. SDG is seen to reduce the incidence of diabetes in a study carried out in rats. A study was performed on diabetic patients with coronary heart disease.They were supplemented with flaxseed oil for three months and the result obtained provided information that the level of gene expression of peroxisome proliferator-activated receptor-gamma (PPAR-γ), lipoprotein (a) LP(a), interleukin-1 (IL-1), and tumor necrosis factor-alpha (TNF-α), but did not influence low-density lipoprotein receptor (LDLR), interleukin-8 (IL-8) and transforming growth factor-beta (TGF-β) [16]. In another study to check the effect of SDG on type 2 diabetes, female rats were administered SDG orally before the onset of diabetes, which delayed the development of diabetes in them by 80%, and the other group of rats that were not treated with SDG developed DM in the same period [17].

Diabetes occurs as a result of oxidative stress by reactive oxygen species (ROS). SDG in flaxseeds can scavenge the role of ROS and ultimately protect the liver from damage, thus preventing the development and worsening of diabetes. High levels of soluble fiber and other bioactive components of flaxseeds help in maintaining normal plasma glucose levels and have a protective effect against diabetes risk by affecting insulin secretion and the mechanism through which insulin performs its function. Flaxseeds also maintain the postprandial blood glucose in an individual. According to a study,young females consuming 50g ground flaxseeds on daily basis for four weeks had resulted in low levels of blood glucose**.** One more study revealed that when postmenopausal women consumed a 40-gram flaxseed fortification diet on daily basis, their blood glucose levels also became normal**.** In a study, two groups were taken. One was given bread that contains 25% of flaxseeds and the other consumed regular bread that did not have flaxseeds. The results showed 28% lower glycemic response in the group consuming flaxseeds-containing bread than the other [18-20].

Another study was performed in 2009 to find the anti-diabetic effect of flaxseeds on non-insulin-dependent diabetes mellitus (NIDDM). Twenty patients were taken in this study and regularly fed chapatti that contains 5g of flaxseed and 25g wheat flour for three months and compared to the control group that was non-diabetic. Results showed a considerable reduction in plasma glucose levels [21].

## Conclusions

Diabetes mellitus is a syndrome that occurs when blood glucose levels rise above normal levels. People who suffer from DM can face polydipsia and polyuria. It is treated by different medications that involve the enhancement of pancreatic beta cells that secrete insulin as well as improve insulin function. Different studies show that diabetes can be prevented and cured by the intake of several foods and food components. Data from different studies recommend that consumption of flaxseeds and sunflower seeds can help in alleviating the effects of diabetes due to their anti-diabetic effects. However, further studies are needed to determine the exact mechanism of the chemicals in these seeds on insulin secretion and insulin resistance in diabetic individuals.
